# A Novel Cre Recombinase Imaging System for Tracking Lymphotropic Virus Infection *In Vivo*

**DOI:** 10.1371/journal.pone.0006492

**Published:** 2009-08-04

**Authors:** Bernadette M. Dutia, Stuart J. Reid, Derek D. Drummond, Yvonne Ligertwood, Ian Bennet, Willard Rietberg, Ondine Silvia, Michael A. Jarvis, Anthony A. Nash

**Affiliations:** 1 Centre for Infectious Diseases, The Roslin Institute and Royal (Dick) School Veterinary Studies, University of Edinburgh, Summerhall, Edinburgh, United Kingdom; 2 Vaccine and Gene Therapy Institute, Oregon Health Sciences University, Portland, Oregon, United States of America; Cambridge University, United Kingdom

## Abstract

**Background:**

Detection, isolation, and identification of individual virus infected cells during long term infection are critical to advance our understanding of mechanisms of pathogenesis for latent/persistent viruses. However, current approaches to study these viruses *in vivo* have been hampered by low sensitivity and effects of cell-type on expression of viral encoded reporter genes. We have designed a novel Cre recombinase (Cre)-based murine system to overcome these problems, and thereby enable tracking and isolation of individual *in vivo* infected cells.

**Methodology/Principal findings:**

Murine gammaherpesvirus 68 (MHV-68) was used as a prototypic persistent model virus. A Cre expressing recombinant virus was constructed and characterised. The virus is attenuated both in lytic virus replication, producing ten-fold lower lung virus titres than wild type virus, and in the establishment of latency. However, despite this limitation, when the sEGFP7 mouse line containing a Cre-activated enhanced green fluorescent protein (EGFP) was infected with the Cre expressing virus, sites of latent and persistent virus infection could be identified within B cells and macrophages of the lymphoid system on the basis of EGFP expression. Importantly, the use of the sEGFP7 mouse line which expresses high levels of EGFP allowed individual virus positive cells to be purified by FACSorting. Virus gene expression could be detected in these cells. Low numbers of EGFP positive cells could also be detected in the bone marrow.

**Conclusions/Significance:**

The use of this novel Cre-based virus/mouse system allowed identification of individual latently infected cells *in vivo* and may be useful for the study and long-term monitoring of other latent/persistent virus infections.

## Introduction

The study of host-virus interactions in individual cell populations *in vivo* is important for the development of disease control strategies. This is particularly the case for viruses which establish life long latent or persistent infections, frequently within low numbers of cells with minimal virus gene expression, often in a number of different cell types. The ability to “mark” infected cells in a readily detectable manner would significantly enhance the study of these virus infections. One approach, which has been investigated for several latent herpesvirus infections including herpes simplex virus, pseudorabies virus and Marek's disease virus involves insertion of a reporter gene such as β-galactosidase or green fluorescent protein within the viral genome [1]–[5]. This technique requires the use of a latency associated promoter to overcome the general repression of viral promoters during latency, including foreign promoters inserted into the viral genome [6]–[9]. Even then, a latency associated promoter may show tissue-specific expression. For example, the HSV latency associated promoter has been shown to drive expression of β-galactosidase long term in sensory neurons, but the promoter is down-regulated in the CNS [10], [11].

An alternative strategy that avoids many of the problems associated with virally encoded reporter genes is to ‘mark’ the infected cell using a reporter gene located within the host genome that is activated upon virus infection. The Cre-recombinase (Cre)/*loxP* site-specific recombination system has been used to mark cells and tissues *in vivo* in nonviral systems investigating cellular developmental patterns and differentiation [12]–[14]. In this system, expression of a constitutively active reporter gene (eg β-galactosidase or enhanced green fluorescent protein, EGFP) is suppressed by the presence of an upstream stop codon flanked by *loxP* sites (floxed). Cre expression results in excision of the floxed stop codon allowing expression of the previously silenced reporter gene. *In vivo*, the reporter gene can be activated by crossing the reporter mouse with a mouse expressing constitutively active or inducible Cre. Expression of Cre from a viral genome similarly offers the potential to mark infected cells. As cells are permanently marked by a single event and the reporter gene is located within the host cell genome, this approach may also circumvent problems associated with virus genome inactivation during latency.

In the present study, we have applied the Cre/*loxP* system to investigate long-term *in vivo* infection with a prototypic latent/persistent gammaherpesvirus, murine gammaherpesvirus 68 (MHV-68, also known as murid herpesvirus 4). MHV-68 is closely related to the human gammaherpesviruses Epstein-Barr virus (EBV) and Kaposi's sarcoma associated herpesvirus (KSHV) [15], [16]. Studies in mice have shown MHV-68 latency to be complex and involve a number of cell types including B cells, macrophages, dendritic cells and lung epithelial cells. In long term infections, the virus appears to reside in memory B cells whereas the contribution of other cell populations to maintaining the infection is not well understood [17]–[19]. The balance between true latent infection, where there is limited gene expression, and persistent infection involving on-going replication also has not been elucidated.

In the present study, we constructed a recombinant MHV-68 expressing Cre and screened a variety of mouse lines expressing a Cre-activatable reporter gene for expression of the reporter following infection. One mouse line, sEGFP7, which has highly accessible loxP sites expressing EGFP in over 90% of haematopoietic cells activated in ovo [20], proved particularly suited for these studies. In sEGFP7 mice, MHV-68 infection (based on EGFP positive cells) was detected in mediastinal lymph nodes (MLNs) up to 80 days post-infection and in the bone marrow at least until 165 days after infection. Because of the high level of EGFP expression in this reporter system, activated cells could be sorted by FACS and virus gene expression analysed. This marker system provides a novel approach for investigating the nature and distribution of virus-infected lymphoid/heamopoeitic cells and the role of these cells in the establishment and maintenance of latent/persistent virus infections.

## Materials and Methods

### Mice

ROSA26 GFP mice (B6;129-Gt(ROSA)26Sor^tm2Sho^ were purchased from the Jackson Laboratory, USA. ROSA26 lacZ mice were supplied by Dr Annemieke IJpenberg, MRC Human Genetics Unit, Edinburgh. C57BL/6 and BALB/c mice were purchased from Harlan UK Ltd (Oxon, UK). The sEGFP7 mouse strain [20] was a gift from Dr Alexander Medvinsky, Institute of Stem Cell Research, University of Edinburgh. All work was carried out under a UK Home Office license according to the Animals (Scientific Procedures) Act 1986.

### Virus infections

Virus working stocks were prepared by infection of BHK-21 cells as previously described [21]. 4–8 week-old mice were anaesthetized with Halothane (Rhone Merieux Ltd, Harlow, Essex, U.K.) and inoculated intranasally with 4×10^5^pfu virus in 40l sterile PBS. At various times after infection mice were killed by CO_2_ asphyxiation and tissues harvested for virus assays or histology.

### Construction of Recombinant Virus

**Figure 1:**
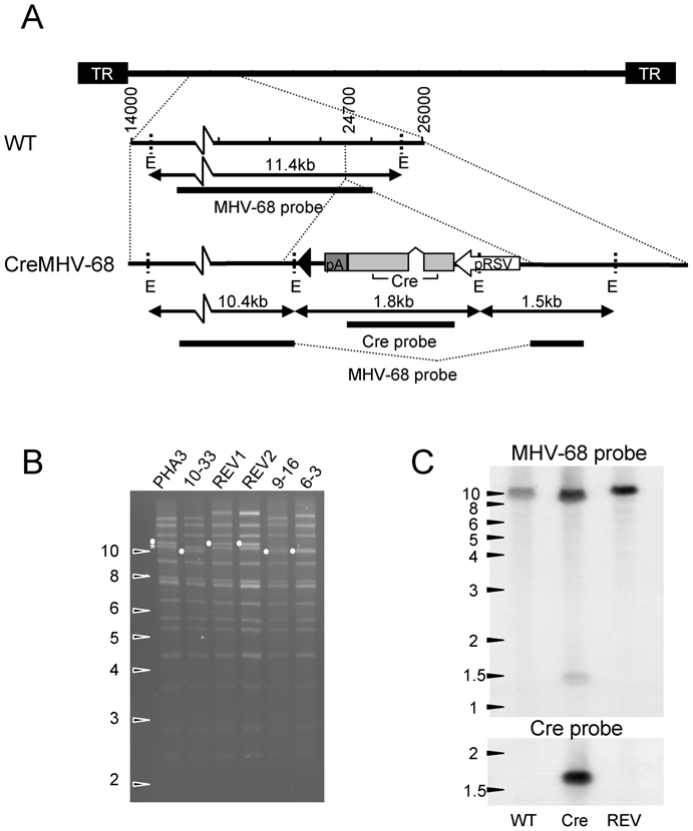
Construction of recombinant MHV-68 expressing Cre-recombinase. A. Structure of the viral genome surrounding the insertion site. Diagram shows the site of insertion, position of restriction sites and probes used for Southern analysis. B. EcoRI digests of BAC DNAs. o 11.4 kb in wild type and revertant reduced to 10.4 in mutant clones. * shows bands containing 100 bp repeats. C. Southern blot of wild type (WT), Cre10–33 and Cre-REV virus DNA digested with EcoRI and probed with an MHV-68 specific probe (nt 21199–25494) and a probe for Cre-recombinase.

Recombinant Cre expressing MHV-68 virus was constructed by E/T recombination using a bacterial artificial chromosome (BAC) containing the entire MHV-68 genome (kindly provided by Dr. U. Koszinowski, Ludwig-Maximilians-Munchen, Munich)[22]. A pcp015-based template plasmid (designated pcp015/Cre) containing the Cre gene under control of the RSV promoter and a 3′ SV40 polyadenylation site was used as a template for generation of the necessary PCR product for E/T recombination. The pcp015/Cre also contains a *FRT*-flanked Kan^R^ marker for selection of recombinants in bacteria (see below); and a synthetic intron within the 5′ coding region of Cre to restrict expression to eukaryotic cells, as initially described by Smith and Enquist [23](Figure 1). E/T recombination was performed essentially as described by Rue [24]. The mutagenic PCR product was obtained from the pcp015/Cre template by PCR amplification using mutagenic primers MHVCre.for (5′-GTGAGTGCTGACAGGCTTAATAAAGAAAATGATTAAATGAAGTAAAACGACGGCCAGT-3′) and MHVCre.rev (5′-GTTGTGTGTAGGAGGTGTGGAAATAAAAACCCTTTAAAATTCAGGAAACAGCTATGAC -3′). These primers contain 41 nt homologous to the MHV-68 sequence flanking the site of insertion within the MHV-68 genome and 17 nt homologous to the pcp015/Cre template. The Cre cassette was inserted within the MHV-68 genome at nt 24700 between ORF11 and K3. E/T recombination was performed in EL250s containing the MHV-68 BAC, and recombinants were selected with chloramphenicol and kanamycin followed by Flp-mediated removal of the *FRT*-flanked Kan^R^ marker. Recombinant MHV-68 BACs were characterized by restriction digestion combined with Southern analysis, as well as direct DNA sequencing of the Cre expression cassette following PCR amplification from the reconstituted viral genome.

Infectious virus was recovered from BAC DNA by Effectene mediated transfection (Qiagen) into BHK cells. The BAC cassette was removed from Cre-recombinant clones by self-excision. BAC DNA was transfected into BHK cells in 6 well plates (Nunc). As soon as colourless plaques (lacking the BAC cassette) were visible, cultures were harvested and replated to limiting dilution in 96 well plates. Wells containing single, colourless plaques were selected and screened by PCR for intact Cre-recombinase.

Revertant BACs were made by allelic exchange using a shuttle plasmid as previously described [22], [25]. The wild type MHV-68 Hind I fragment (nt21965–26711) was cloned into the shuttle plasmid pST76_SR and electroporated into DH10B cells containing the Cre10–33 BAC. Transformed bacteria were then propagated through a multi-step selection process and wild type BAC was selected by PCR. Virus stocks were produced by transfection of BAC DNA into BHK cells. BAC sequences were excised from the reconstituted virus by passage through mouse NIH 3T3 cells expressing Cre recombinase [26].

### Southern Blot Analysis

Viral DNA was prepared from purified virions as previously described [27]. 10 µg of viral DNA was digested with EcoRI, fractionated on a 0.7% w/v agarose gel and transferred to nylon membrane (Hybond^™^ -N+, GE Healthcare Ltd). The blot was probed with [P^32^]dCTP labeled probes for MHV-68 (nt 21199–25494) and Cre-recombinase. Hybridisation was carried out in Ultrahyb (Ambion) according to manufacturer's directions and radioactivity detected by exposure to ECL film.

### Growth of virus *in vitro*

Single-step and multi-step growth *in vitro* was analysed by infection of BHK-21 cells in suspension for 90 minutes at a multiplicity of infection 5 (single-step growth) and 0.05 (multi-step growth). Cells were washed with Glasgow's medium four times to remove unbound virus before seeding into 24-well plates. At specific times post-infection cells were harvested and infectious virus was determined by plaque assay. All infections were carried out in duplicate, and each infection titrated in duplicate.

### Analysis of Infected Tissues

Tissues were analysed for infectious virus by plaque assay and latent infection by infective centre assay as previous described [28]. For histological analysis, tissues were harvested into 4% buffered paraformaldehyde and frozen as previously described to preserve EGFP fluorescence [29].

### Immunohistochemistry

5–10 µm sections were cut onto poly-L lysine coated slides and stored at −70^°^C until required. Sections were blocked for 1 hour at room temperature with 10% normal goat serum in Tris-buffered saline and stained with primary antibodies in 2% NGS for 1 hour at RT. Antibodies used were as follows: CD45R/B220 (RA3-6BS, Pharmingen) to detect B cells, MOMA-1 (AbDSerotec) for metallophilic macrophages, MOMA-2 (AbDSerotec) and F4/80 (CI:A3-1, AbDSerotec) for macrophages. Secondary antibody was goat anti-rat alexafluor 594 (Molecular Probes). Nuclei were stained with TOPRO3 (Molecular Probes). Sections were mounted in Moliol and imaged with a Leica TCS-NT confocal microscope.

### FACS analysis and sorting

Lymphocytes were teased from mediastinal lymph nodes, resuspended in FACS buffer (PBS, 1%BSA, 0.1% sodium azide) and stained with rat anti-mouse CD19 phycoerythrin (Caltag) and hamster anti-mouse CD69 Tricolor (Caltag). Cells were gated on EGFP positive lymphocytes and double stained populations were sorted on a FACS Vantage with DiVa option (BD Biosciences).

### PCR

For analysis of transcription of ORF11 and K3 *in vitro*, C127 murine epithelial cells were infected at a multiplicity of infection of 5 and incubated for 18 hr at 37^°^C. Monolayers were washed twice with ice-cold PBS and RNA was extracted with RNAwiz (Ambion) according to the manufacturer's instructions. Contaminating DNA was removed by treatment with DNAseI (DNA-Free, Ambion). For analysis of sorted populations DNA and RNA were extracted from sorted EGFP positive cells using the PicoPure DNA and the PicoPure RNA extraction kits (Arcturus) respectively according to the manufacturer's instructions. cDNA was prepared from 5 µg RNA or, for the sorted populations, total RNA recovered and PCR was carried out as previously described [30] using the following primers: K3F 5′- GGGTATCAGGACAAG GGGTAG -3′, K3R 5′- GAGCTACTACACTACCTCCTG -3′; ORF11for 5′- GGTGGA CTTTAAGCCCGATG -3′; ORF11rev 5′ –GGC AGC TTC ACT GAC ACC AG 3′; ORF73FOR: 5′- TAGATCCAGGTGATCCTGTGGC -3′; ORF73REV: 5′- CCGCATAATCCATCTGATCCAT -3′). PCR products were fractionated on an agarose gel and visualized with ethidium bromide.

For quantitation of viral load *in vivo* DNA was prepared from mediastinal lymph node cells harvested at day 5 post-infection using the PureLink Genomic DNA Purification Kit (Invitrogen). Real-time PCR for quantification of viral genome load was carried out on 100 ng lymphocyte DNA using a Rotor-Gene (Corbett) with the intercalating dye SYBR green. Regions of the M11, ORF73 and M4 genes were amplified with specific primers (ORF73QPCRfor 5′-CGTCTGTCTCTCCTACATCTAAACC-3′; ORF73QPCRrev 5′-CACCAACACTTCCCTCATCC-3′; M4-RTFor 5′-CAC CTG AGA TCA AGT CTA TCG-3′; M4-RTRev 5′-GTC GCA TAA CCA TGT CCA CG-3′) and all products analysed by a melt curve to confirm specificity. DNA load was quantitated using cloned DNA and results were normalized with a GAPDH real-time PCR.

## Results

### Construction of MHV-68 virus expressing Cre

To enable detection of virus infected cells *in vivo*, a virus expressing a modified intron containing Cre-recombinase [23] was constructed by mutagenesis of a MHV-68 bacterial artificial chromosome (BAC) [22]. Linear recombination was used to insert a cassette containing Cre under control of the RSV LTR into the MHV-68 genome between the ORF11 and K3 genes at nt24700 (Figure 1A). Three independent clones designated 6–3, 9–16 and 10–33 were selected. Correct insertion of the Cre expression cassette within the MHV-68 BAC genome was confirmed by restriction digestion of BAC DNA (Figure 1B) followed by Southern analysis, as well as by direct DNA sequence analysis of a PCR product spanning the insertion site (data not shown). Revertant BAC was constructed by allelic exchange and confirmed as detailed above for Cre recombinant clones. Because BAC recombination can result in loss of internal repeats, recombinant BAC DNA preps were digested with EcoRI and monitored for changes in repeat fragments. One clone, 6–3, showed a reduced number of 100 bp repeats and therefore was not used for further analysis. No other differences in repeats between the recombinant clones and the parent were detected by this technique although all the BAC clones seem to have lost their 40 bp repeat (Figure 1B). Virus was reconstituted by transfection of BAC DNA into MHV-68 permissive BHK cells. The presence of BAC sequences has been shown to attenuate reconstituted MHV-68 virus [31]. Therefore BAC sequences were removed from Cre expressing clones by self-excision in BHK cells, indicating that the RSV LTR promoter is active during lytic infection or by passage through a Cre-expressing cell line (revertant). Once the BAC cassette was removed, the viral genome was stable. Restriction digest followed by Southern blot analysis of purified viral DNA was carried out to confirm the genotype of the virus. Figure 1C shows that the expected digestion pattern was observed. Virus stocks were monitored by PCR to confirm the genotype routinely.

### Characterisation of recombinant virus *in vitro*

**Figure 2:**
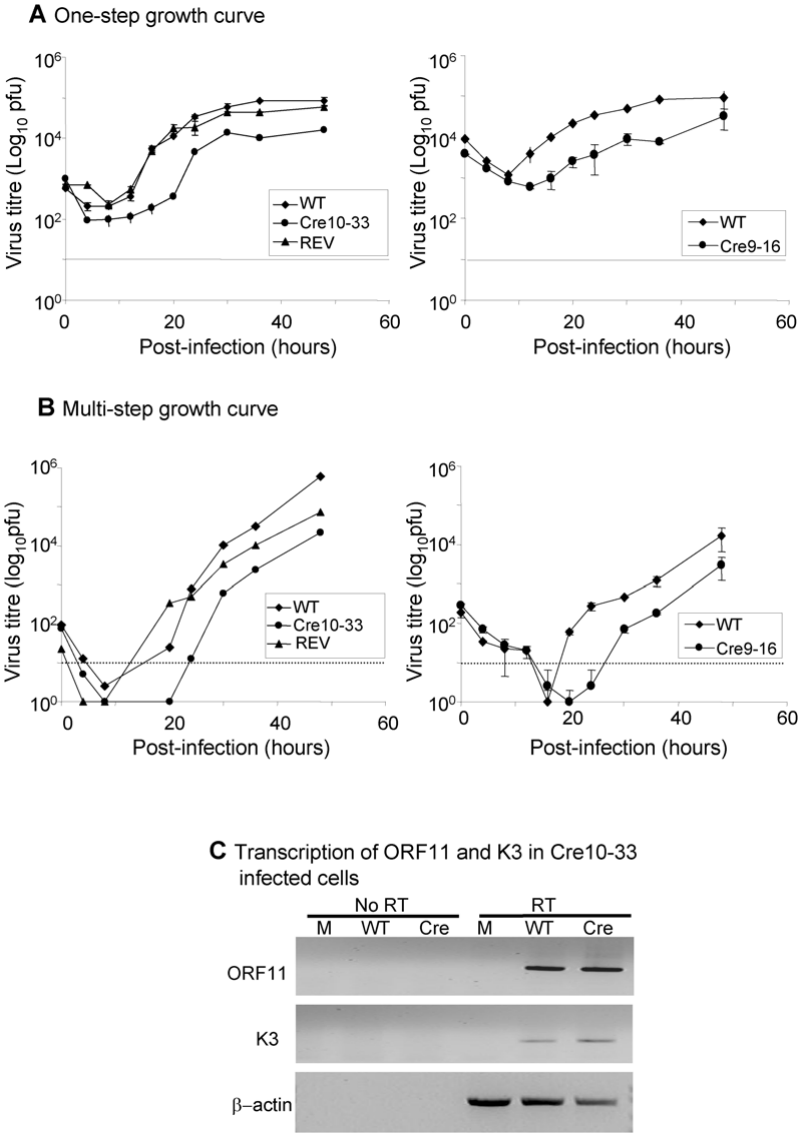
*In vitro* characterisation of CreMHV-68 virus. To compare replication of Cre expressing MHV-68 clones 10–33 or 9–16 with wild type PHA-4 and a revertant of 10–33 Cre-REV virus, BHK cells were infected with 5pfu/cell (single-step, A) or 0.05pfu/cell (multi-step B) and titred after harvesting at specific time points post-infection. The data represent the mean +/− the standard deviation. C. RT-PCR for detection of K3, ORF11 or β−actin transcripts in C127 cells infected for 18 h with wild type (WT) or CreMHV-68 (Cre) or mock-infected.

In order to determine whether insertion of the Cre-recombinase cassette affected growth of the recombinant virus, replication of the Cre expressing viruses 9–16 and 10–33 was compared with wild type (WT) BAC derived virus, PHA-4 and a revertant constructed from the 10–33 BAC clone (REV) *in vitro*. Figure 2 shows single- and multi-step analysis of the viruses. The kinetics of the one- step growth curve showed a delay in the growth of 10–33 and 9–16 when compared with the revertant or wild type MHV-68. Both clone 9–16 and clone 10–33 also showed delayed growth at early times in the multi-step analysis.

To determine whether insertion of the Cre expression cassette affected transcription of the surrounding genes, C127 cells were infected with WT or Cre10–33 and expression of K3 and ORF11 which flank the Cre insertion site was analysed by RT-PCR. Figure 2C shows no difference in transcription of K3 and ORF11 in the Cre10–33 compared to WT-infected cells.

### Characterisation of recombinant virus *in vivo*

**Figure 3:**
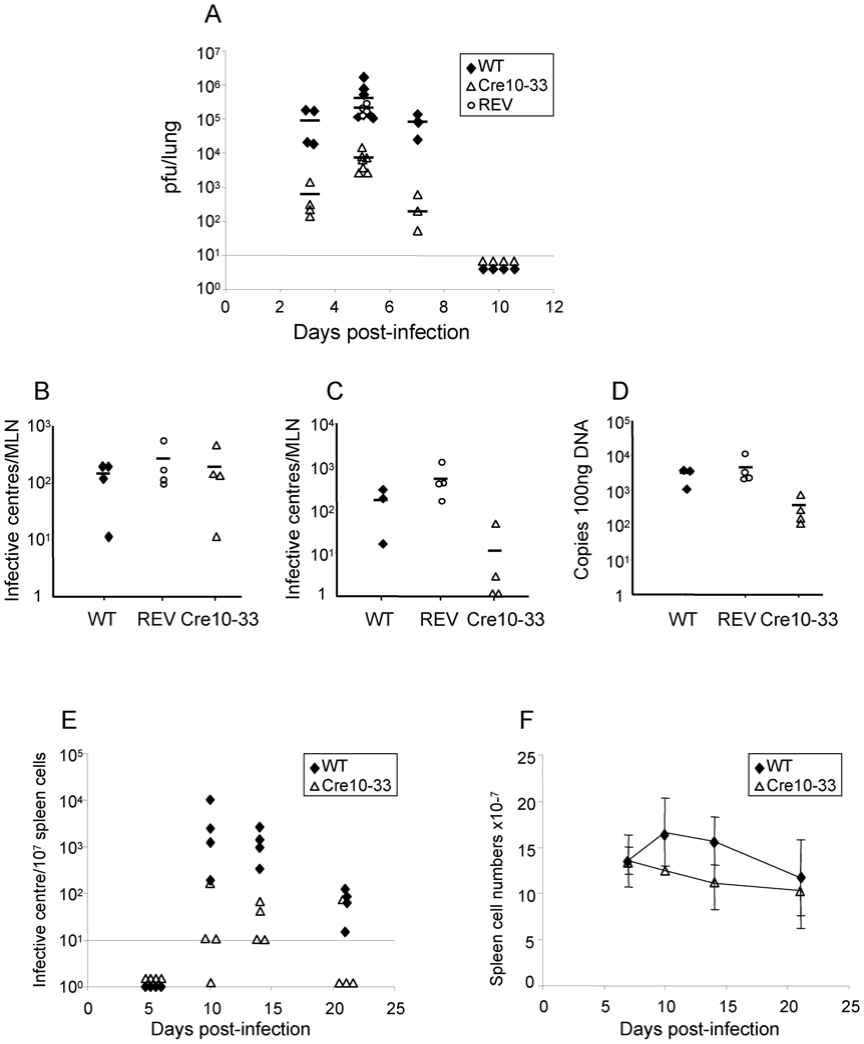
*In vivo* characterisation of CreMHV-68 virus. BALB/c mice were infected intranasally with wild type MHV-68 (WT), Cre-recombinase expressing MHV-68 (Cre10–33) or revertant virus (REV). Tissues were removed at various times and assayed for infectious virus by plaque assay, for latent virus by a reactivation assay and for viral DNA by Q-PCR. A. Infectious virus in the lung; B. Latent virus in mediastinal lymph node (MLN) at day 5; C. Latent virus in the MLN day 14; D. Viral DNA load in MLN at day 14; E. Latent virus in the spleen; F. Spleen cell numbers. Solid line indicates mean.

Growth of viruses *in vivo* was assessed by intranasal infection of BALB/c and C57Bl/6 mice. Similar results were observed with both strains of mice and initial results showed that viruses derived from 9–16 and 10–33 behaved identically. All further analysis was therefore carried out with 10–33 derived virus (Cre10–33). Cre10–33 replicated within the lung, although virus titres were consistently lower than those obtained with wild type or revertant virus (Figure 3A). The Cre10–33 virus was cleared with similar kinetics to WT virus. Following replication in the lung, MHV-68 establishes latent infection in the lymphoid system, initially within the mediastinal lymph node where virus is associated with macrophages, dendritic cells and B cells. The virus undergoes a B cell-associated viraemia to infect other tissues such as the spleen where the latent infection resides in the germinal centres. Latent infection in lymphoid tissues is accompanied by lymphoproliferation.

Levels of latent virus in the mediastinal lymph nodes (MLN) were determined by reactivation assay and quantitative PCR. Assays for pre-formed infectious virus were carried out to confirm that the reactivation assay was detecting latent virus and any infectious virus was subtracted from the figures obtained in the latency assay. In the majority of samples, infectious virus (Cre10–33, WT or REV) was not detected in the MLNs. When infectious virus was detected, the levels were always <10% of the values of latent virus. Five days pi, the number of latently infected cells in the MLNs ranged from 12–634 cells per total lymph node for WT and REV infected mice and 12–488 cells/MLN for the Cre 10–33 infected mice (Figure 3B). These data indicate that the initial phase of latency established in Cre10–33 infected mice is similar to that in mice infected with WT and REV.

However, by day 14, the Cre10–33 viral DNA load in the MLN was around 10 fold lower than in WT or REV infections (Figure 3D) and there few reactivatable cells present in the MLN (Figure 3C; 20–1340 infective centres per MLN for wild type and REV vs 0–50 for Cre10–33). These results are consistent with failure of Cre10–33 infected cells to amplify to the same extent as WT infected cells.

Analysis of latent infection in the spleen by reactivation assay (Figure 3E) showed that Cre10–33 could not establish latent infection at the same level as wild type at any time point and resulted in lower levels of lymphoproliferation (Figure 3F).

These experiments indicate that insertion of the Cre-recombinase cassette into MHV-68 has resulted in attenuation of the virus. However, the virus is clearly capable of establishing latent infection in the lymphoid system and therefore provides a useful tool to determine whether virus encoded Cre-activation of a floxed reporter in mice is able to identify latently infected cells *in vivo*.

### Tracking virus infection of cells in the lymphoid system

**Figure 4:**
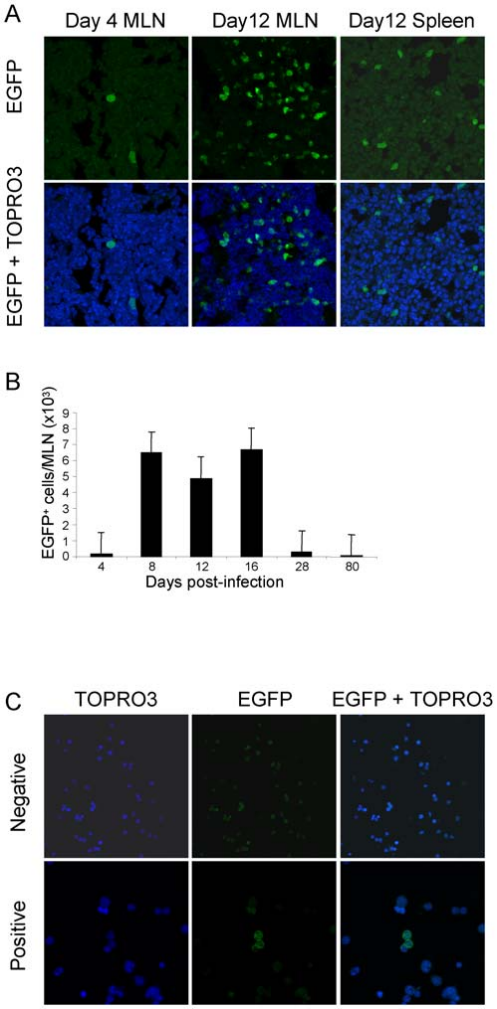
Detection of EGFP positive cells *in vivo*. A. EGFP positive cells in MLN and spleen of sEGFP7 mice infected with Cre10–33. Top panels show EGFP only, bottom panels, EGFP plus TOPRO3 nuclear staining. B. Total number of EGFP positive cells in MLNs over the course of infection. Cell numbers determined by microscopy. Error bars represent standard deviations from mean. C. EGFP positive cells in bone marrow 165 days post-infection. Images obtained with a Leica confocal microscope. Magnification×40, negative; ×63, positive.

In initial *in vivo* studies using Cre10–33, we were consistently unable to detect EGFP or *lacZ* expressing cells by fluorescent imaging or β-gal staining of infected ROSA26 EGFP or *lacZ* Cre reporter mouse strains (data not shown). However, following infection of the sEGFP7 mouse line [20] EGFP was readily detected in MLN and spleen at various time points after infection. This mouse strain expresses EGFP under control of the PGK promoter. Expression has been studied in detail in the haematopoetic tissues and shown to be high level, readily detected by FACS in all cell types and stable over long periods of time. sEGFP7 mice were infected with Cre10–33 or WT virus, and at days 4, 8, 12, 16, 28 and 80 pi MLNs were removed, frozen and 10 µm cryostat sections were prepared from each entire lymph node. Figure 4A shows confocal images of EGFP positive cells in representative sections at day 4 and day 12 pi. The sections were examined by UV light microscopy and EGFP positive cells were counted (Figure 4B). On day 4 pi, an average of 70 EGFP positive cell was detected/lymph node. By day 8 post-infection, the average number of positive cells/lymph node rose to over 6000, and remained at this elevated level (4000–6000 cells) on days 12 and 16 pi. At later time points the average number of EGFP positive cells/lymph node decreased and ranged from 50 to 150. Importantly, EGFP positive cells were still detectable 80 days pi. Thus, although the numbers of cells in the MLN harbouring Cre10–33 virus, as determined by the reactivation assay, appeared to be low at 2 weeks pi (<50 infective centres, see Figure 3C), the large number of EGFP positive cells indicates that a latent infection was readily detected at this time (Figure 4B), reinforcing our conclusion that Cre10–33 can establish a latent infection.

From initial replication in the MLN, MHV-68 is believed to be disseminated to other tissues by infected B cells. Consistent with this model, EGFP positive cells were detected in the spleen at day 12 (Figure 4A) and at subsequent times pi (data not shown). To determine whether virus infected cells could be detected outside secondary lymphoid organs, cytospins prepared from bone marrow at days 16, 28, 80 and 165 were screened from EGFP positive cells. Small numbers of EGFP positive cells (<1 in 10^6^) were detected at day 28 (Figure S1) and were still detectable at day 165 pi (Figure 4C). Together these results show that the system provides a powerful method of tracking virus infection in different tissues *in vivo*.

### Phenotyping of EGFP positive cells

**Figure 5:**
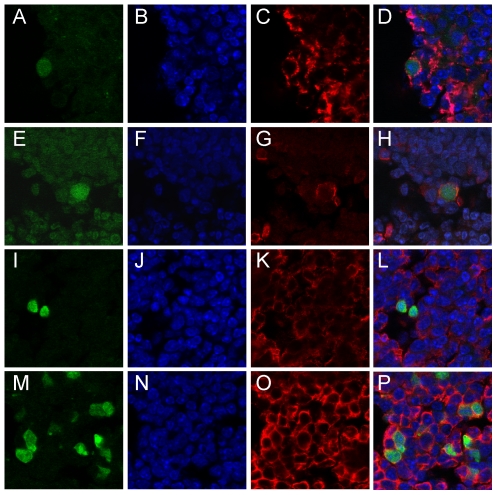
Phenotype of EGFP positive cells in the MLN of sEGFP7 mice infected with Cre10–33. A-H day 4; I-P day 12. A-D show images of MOMA1 positive macrophage, E-H and M-P show detection of B220 positive B cells; I-L show macrophages stained with a combination of anti-macrophage antibodies, MOMA1, MOMA2 and F4/80. Sections imaged with a Leica confocal microscope. Magnification×63.

The phenotype of EGFP positive cells in the MLNs of Cre10–33 infected sEGFP7 mice was determined by confocal microscopy of cryostat sections stained with antibody to B cells (B220) or a mixture of anti-macrophage antibodies (MOMA1, MOMA2 and F4/80) capable of detecting several macrophage subtypes. Figure 5 shows that EGFP positive B cells and macrophages were readily detected at days 4 and 12pi providing direct visual evidence for infection of these cell types and opening up the possibility of selecting specific infected cells for further study.

### Isolation of EGFP positive cells by flow cytometry

**Figure 6:**
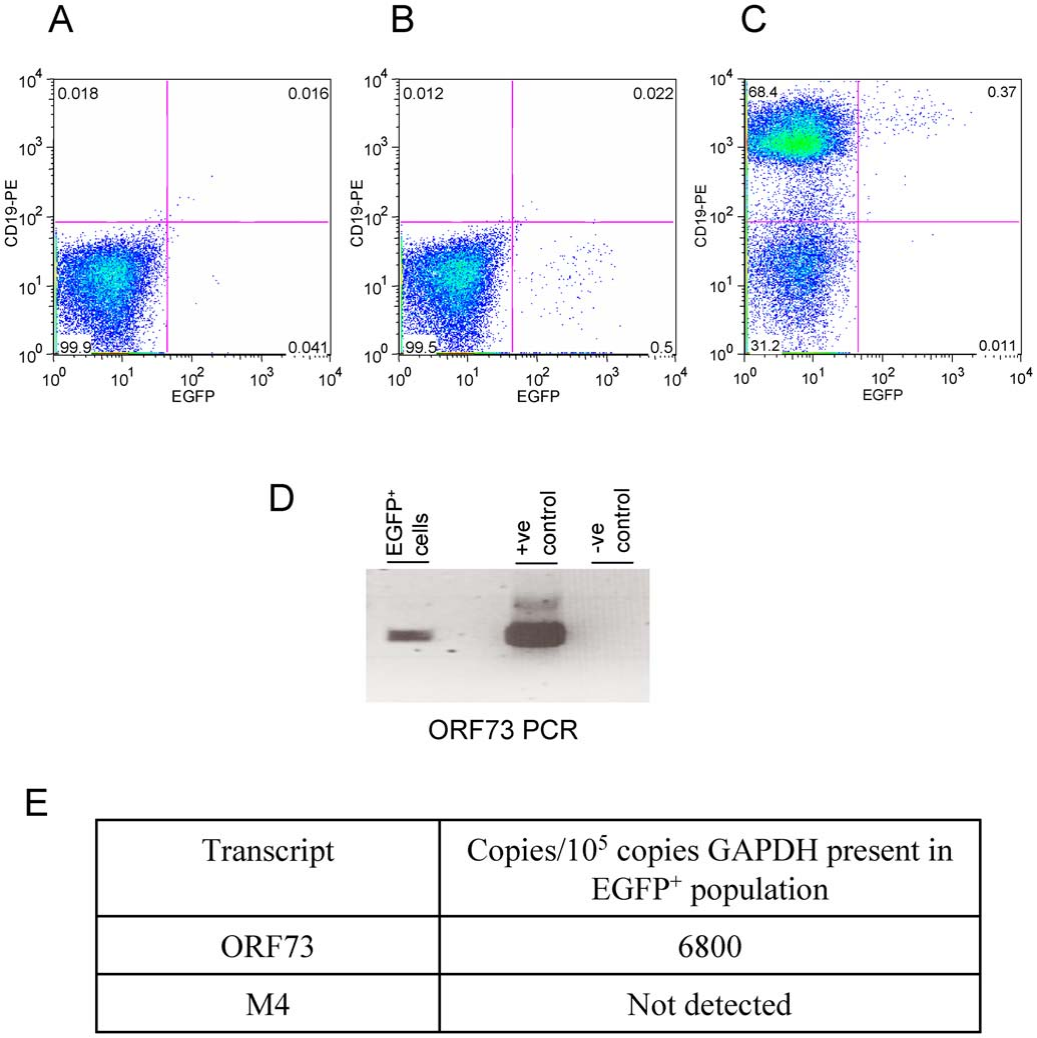
FACS analysis of lymphocyte population of MLNs from sEGFP7 mice 16 days after infection. A. sEGFP7 mouse infected with wild type virus (negative control); sEGFP7 mice infected with Cre (10–33) unstained (B) or stained (C) with CD19-PE; D. DNA from sorted EGFP positive cells was subjected to PCR using primers for MHV-68 ORF73 together with positive (MHV-68 DNA) and negative (H_2_O) controls. E. Quantitative RT-PCR for viral transcripts in EGFP^+^ CD19^+^, CD69^+^ cells. When quantitative PCR was carried out on equivalent amounts of DNase treated RNA, GAPDH was undetectable and copy numbers of ORF73 were <0.05 of those of cDNA.

The large number of positive cells present in the MLNs at days 12–16 pi suggested that isolation of virus positive cells by FACSorting might be possible. The ability to select virus infected cells in this way would make analysis of virus and host transcriptomes in specific cell populations readily obtainable. Therefore, MLNs from sEGFP7 mice infected with Cre10–33 or WT were analysed for EGFP expression. Figure 6 shows that it was possible to detect EGFP positive cells in the MLNs of Cre10–33 infected sEGFP7 mice by FACS analysis. In an analysis of individual MLNs from 5 mice at day9 and 15 mice at day16, the mean percentage of EGFP^+^ cells was 0.25% of the gated lymphocyte population and <0.2% of the total population. Up to 19000 EGFP+ cells could be isolated from an individual MLN. Representative data are shown in Table 1. Double staining of MLNs for the B cell marker CD19 showed that the majority of the EGFP positive cells were B cells (Figure 6A-C). EGFP cells were isolated by FACSorting, DNA and RNA were extracted and analysed by PCR and RT-PCR for the presence of virus genome and transcripts. Figure 6D shows that viral DNA was detectable in the EGFP positive population and the latency associated transcript ORF73 was readily detected.

**Table 1:** Percentages of EGFP^+^ cells in mediastinal lymph nodes and numbers of cells recovered from total lymph node by FACSort.

Mouse number	Day post-infection	% lymphocytes EGFP^+^	Number of cells sorted/MLN
1	9	0.1	10000
2	9	0.1	4231
3	9	0.1	3959
4	16	0.1	2800
5	16	0.1	3704
6	16	0.1	2399
7	16	1.6	18938
8	16	0.1	5742
9	16	0.1	10397
19	16	0.1	2107

Activated B cells were selected by FACSorting of EGFP^+^, CD19^+^, CD69^+^ cells from MLNs at day16 pi. Analysis of these cells by quantitative RT-PCR demonstrated the presence of the latency associated transcript ORF73 (Figure 6E) but expression of the lytic cycle gene M4, which is more readily detectable than ORF73 in lytically infected cells [32] could not be detected thus providing evidence for latent infection in this cell population. The system thus provides a powerful means of isolating individual infected cell populations and potentially enabling fundamental questions to be addressed.

## Discussion

We have developed a novel system to detect and characterise virus-infected cells *in vivo*. The system exploits a Cre-expressing virus and mice containing EGFP under the control of a *floxed* transcription stop codon. MHV-68 was used to model infection of the lymphoid system enabling fundamental questions to be addressed on the kinetics of latent infection, the detection of novel sites of latency and the potential to explore host and virus gene expression in individual infected cells at different stages following infection. A key feature of this model was the availability of the sEGFP7 mouse strain as other mouse strains investigated did not allow detection of infected cells.

The Cre-loxP system has been used to identify neurons latently infected with HSV. This involved a Cre expressing HSV and the ROSA26R mice carrying a *lac*Z reporter [33]. In our model we were unable we were unable to detect *lac*Z expression in the lymphoid system of ROSA26R mice, possibly reflecting lower promoter activity in this tissue. Further, the use of an EGFP reporter has the advantage that live cells can be detected and sorted without the need to carry out *ex vivo* staining. A related approach relying on tissue specific expression of Cre-recombinase and an EGFP gene preceded by a “floxed” stop codon inserted into the MCMV genome has been used to track lytic infection *in vivo*. In this system, lytically infected cells express EGFP and progeny virus permanently encode activated EGFP gene. Thus the contribution of different cell types to lytic virus replication and spread of infectious virus can be tracked. This system, however, unlike the system described here does not mark latently infected cells [34].

The presence of the Cre cassette clearly attenuates the virus both *in vivo* and *in vitro*. *In vivo* the virus replicates less efficiently in the lung and, whereas the early stages (day 5) in the establishment of a latent infection are similar to wild type virus, by day 14 the number of latently infected cells as determined by the infective centre assay is greatly reduced and the viral DNA loads are around 10 fold less than wild type. These data together with the numbers of EGFP^+^ cells in the MLN at day 16 measured by both FACS and microscopic analysis are consistent with a failure of the virus to amplify to the same levels as wild type virus.

The reason for the attenuation is not clear. The RSV LTR is active during lytic infection, as shown by the ability of the BAC to self-excise, and the ability to detect EGFP expression in the MLN where the predominant infection is latent strongly suggests it is active during at least the early stages of latent infection. It is possible that RSV promoter activity within the virus genome alters the activity of viral promoters. Consistent with this idea, the insertion of the *lacZ* under the human cytomegalovirus immediate early promoter has been shown to alter the replication of MHV-68 *in vivo* [35]. Downstream effects from the RSV promoter could affect virus replication both *in vitro* and *in vivo*. Attenuation of virus replication in the lung and latent virus load in the spleen may be related to EGFP expression although the sEGFP7 line is on a C57Bl/6 background and EGFP has been reported to have a minimal immunogenic effect in this mouse strain [36]. In preliminary experiments we have found that depletion of CD8 T cells in Cre10–33 infected sEGFP7 mice results in 100×higher levels of reactivatible virus in the spleen at days 12 and 20. It is possible, therefore, that the RSV promoter is driving expression of a virus gene which is a CD8 target.

Although the virus is attenuated, it can to establish a latent infection. Within the MLN, the number of infected cells increased over the first week reaching a plateau around day 8 and remained elevated for a further 8 days. By 4 weeks post-infection, the number of infected cells had decreased. The kinetics of latent infection are similar to those demonstrated by infective centre assays as a measure of reactivation from latency and by Q-PCR as a true measure of cells harbouring viral DNA. The method defines an ‘acute phase’ of latency in which there is a rapid expansion of latently infected cells that act as a reservoir for seeding by the vascular system to other tissues. In this model, EGFP positive cells are detected in the spleen by day 12 post-infection and in the bone marrow there are infected cells present from day 28 to at least 165 days post-infection. The bone marrow has been identified as a site of latent infection [37] but the nature of the infected bone marrow cell has not been determined. This technique offers the potential for identifying the infected bone marrow cells and for detecting and identifying other novel infected cell types.

EGFP^+^ cells in the MLN can be selected by FACS presenting a powerful approach to investigate in detail transcription patterns in cells of different phenotypes at different time points during the course of infection. We have sorted EGFP^+^ CD19^+^ CD69^+^ cells and quantified ORF73 and M4 expression in this population providing evidence that the cells are latently infected. Analysis of virus gene expression in B cell subsets isolated by FACSorting has shown that, 14 days pi, virus gene expression is restricted and depends on the differentiation stage of the B lymphocyte [17]. The technology we describe offers the potential to enhance and extend these studies using pure populations of virus infected cells at time points where the proportion of virus infected cells within the sorted cell populations is very low. This will be particularly important in analysing small numbers of latently infected cells situated in different tissue compartments, where a variable state of latency may exist.

In summary, the application of Cre-*loxP* technology provides new opportunities to study the molecular basis of persistence/latency in cells of the immune system identified at different times after infection. Furthermore, one important application of this approach is the discovery of new cell types associated with persistent/latent virus infection.

## Supporting Information

Figure S1 EGFP postive cells in bone marrow 28 days post infection. Images obtained with a Leica confocal microscope. Magnification×63.
(1.95 MB TIF)
