# Supplementary figures and images for: A Novel Cre Recombinase Imaging System for Tracking Lymphotropic Virus Infection In Vivo
Source: PLoS One. 2009 Aug 4;4(8):e6492. doi: 10.1371/journal.pone.0006492 (PMC2714982; doi:10.1371/journal.pone.0006492)

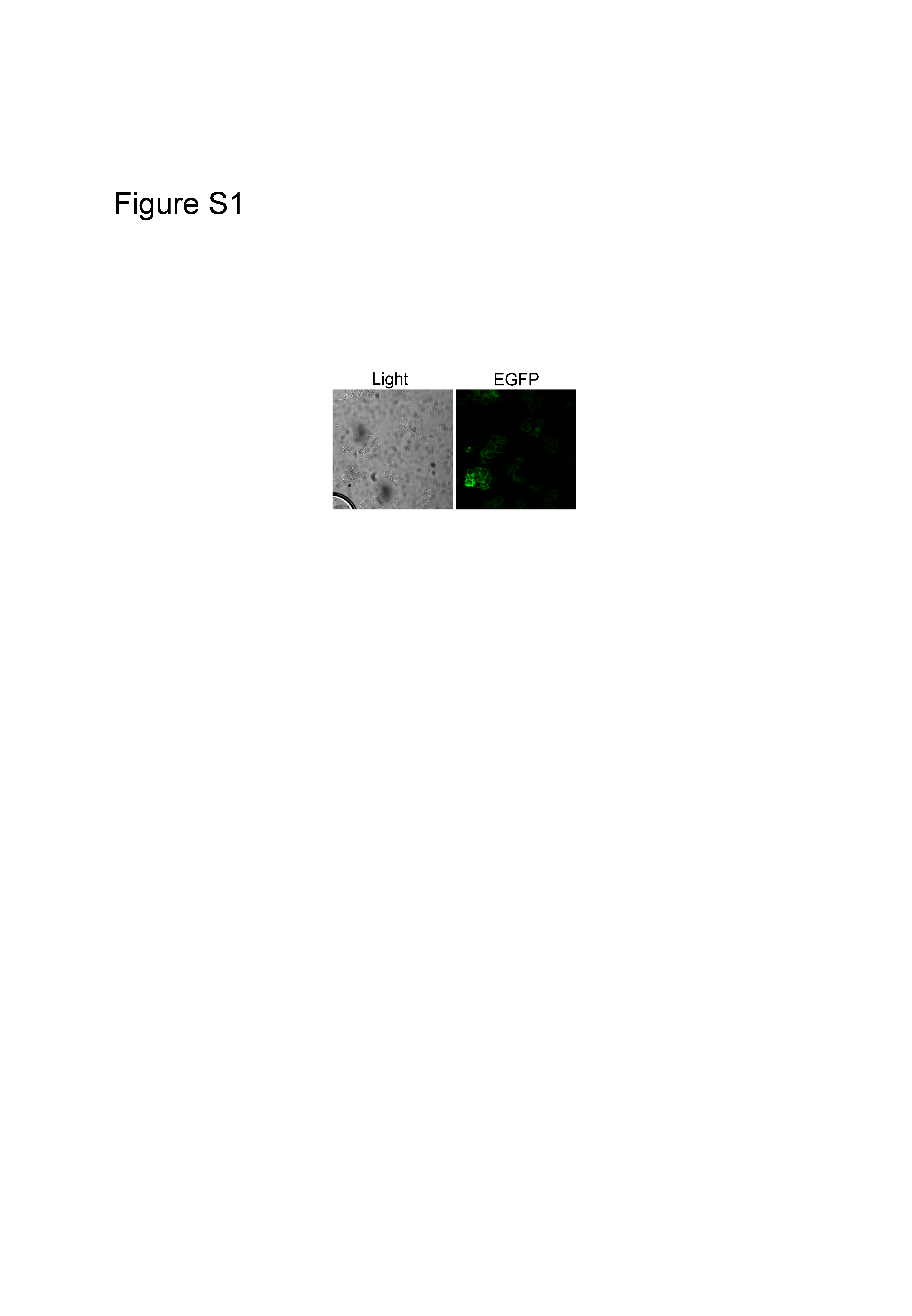

Supplement: Figure S1 — EGFP postive cells in bone marrow 28 days post infection. Images obtained with a Leica confocal microscope. Magnification×63. (1.95 MB TIF) [file pone.0006492.s001.tif]
